# Mobile Jump Assessment (mJump): A Descriptive and Inferential Study

**DOI:** 10.2196/rehab.4120

**Published:** 2015-08-26

**Authors:** Alvaro Mateos-Angulo, Alejandro Galán-Mercant, Antonio Cuesta-Vargas

**Affiliations:** ^1^Universidad de MalagaInsituto de Investigación Biomedica de MalagaMalagaSpain

**Keywords:** squat jump, countermovement jump, inertial sensor, smartphone

## Abstract

**Background:**

Vertical jump tests are used in athletics and rehabilitation to measure physical performance in people of different age ranges and fitness. Jumping ability can be analyzed through different variables, and the most commonly used are fly time and jump height. They can be obtained by a variety of measuring devices, but most are limited to laboratory use only. The current generation of smartphones contains inertial sensors that are able to record kinematic variables for human motion analysis, since they are tools for easy access and portability for clinical use.

**Objective:**

The aim of this study was to describe and analyze the kinematics characteristics using the inertial sensor incorporated in the iPhone 4S, the lower limbs strength through a manual dynamometer, and the jump variables obtained with a contact mat in the squat jump and countermovement jump tests (fly time and jump height) from a cohort of healthy people.

**Methods:**

A cross sectional study was conducted on a population of healthy young adults. Twenty-seven participants performed three trials (n=81 jumps) of squat jump and countermovement jump tests. Acceleration variables were measured through a smartphone’s inertial sensor. Additionally, jump variables from a contact mat and lower limbs dynamometry were collected.

**Results:**

In the present study, the kinematic variables derived from acceleration through the inertial sensor of a smartphone iPhone 4S, dynamometry of lower limbs with a handheld dynamometer, and the height and flight time with a contact mat have been described in vertical jump tests from a cohort of young healthy subjects. The development of the execution has been described, examined and identified in a squat jump test and countermovement jump test under acceleration variables that were obtained with the smartphone.

**Conclusions:**

The built-in iPhone 4S inertial sensor is able to measure acceleration variables while performing vertical jump tests for the squat jump and countermovement jump in healthy young adults. The acceleration kinematics variables derived from the smartphone’s inertial sensor are higher in the countermovement jump test than the squat jump test.

## Introduction

In athletics and rehabilitation, functional capacity can be evaluated through different methods; one of them is the vertical jump test. In addition, the vertical jump serves as a predictor of anaerobic capacity, motor development and athletic ability in sports [1-7]. Other studies report that the vertical jump test seems to be an indicator for assessing functional capacity in the elderly [8] and children [9]. Vertical jump capacity can be measured by different variables such as vertical take-off speed, flight time, mechanical power or the displacement of the center of body mass [10-13].

Some of the classic measurement tools to calculate these variables are the force platform, video-analysis systems, photoelectric cells and contact mats [10-13]. These methods for vertical jumping measurement have excellent validity for laboratory studies. However, these tools are expensive and difficult to transport, creating difficult transferability to other environments or professional applications. Furthermore, in recent years new technologies have begun to be used for human motion studies, such as inertial sensors, which are small and portable, and provide solutions to the drawbacks of other commonly used instruments for human motion analysis [14]. In some studies, accelerometric systems have been used to estimate vertical jump capacity [15-19]; these are based on the use of acceleration peak values recorded in the performance of vertical jumps.

Currently, the latest mobile phone generation usually includes inertial sensors with subunits such as accelerometers and gyroscopes that can detect acceleration and the inclination of devices. Numerous apps that display, store and transfer inertial sensor data have been developed for the operation of different mobile phones. These apps have great potential for tracking human motion parameters for research and clinical practice. Apps are being developed for use in different situations related to human movement, such as the pedometer [20], or the development of an assessment tool and quantification of kinematic variables related to the fragility of the elderly [21]. The wide availability of mobile phones, due to the variety of use in the daily lives of most people in developed countries, as well as their small size and portability, make them very useful tools for field study development and subsequent use in professional practice [14].

Because of the advantages offered by mobile phones as tools for the study and analysis of human movement, it is of interest to check their ability to assess and analysis vertical jump tests. The aim of this study was to describe and analyze kinematics characteristics using the inertial sensor incorporated in the iPhone 4S, lower limb strength through a manual dynamometer, and the jump variables obtained with a contact mat in the squat jump (SJ) and countermovement jump (CMJ) tests from a cohort of healthy people. The squat jump (SJ) is defined as a jump that is performed from a squatting position. A counter movement jump (CMJ), which is higher, is a jump where the jumper starts from an upright standing position, makes a preliminary downward movement by flexing at the knees and hips, then immediately jumps up from that position. 

##  Methods

### Design and Participants

This was a cross-sectional study, involving 81 jumps from 27 participants. The participants were young Health Sciences students from the University of Málaga (Spain). Participants had to meet the inclusion criteria of being healthy adults aged 18 to 35 years, without musculoskeletal or neurological dysfunction. Subjects with any of the following criteria were excluded: a history of heart disease, surgical interventions in the last year, any disability that would make the correct achievement of the tests difficult, any pain that prevented the completion of tests or neuromuscular pathology that could be aggravated by participating in the study. A physical therapist evaluated the volunteers for the presence of exclusion criteria. Table 1 shows the characteristics of the sample.

The study complied with the principles laid out in the Declaration of Helsinki. The ethics committee of the Faculty of Health Sciences at the University of Malaga, Spain, approved the study.

**Table 1:** Characteristics of sample (N=81 jumps).

Characteristic	Mean (SD)
Age (years)	24.30 (3.90)
Height (cm)	173.59 (9.74)
Weight (kg)	72.58 (13.01)
Body mass index (kg/m^2^)	23.95 (2.96)

### Data Collection and Procedures

#### Overview

Study subjects performed three trials of the jump tests described by Bosco [22]: SJ and CMJ (with arm swing modality). The SJ is a maximum vertical jump starting from the position of leg flexion 90°, with no rebound or counter movement and with hands on hips from the beginning to the end of the jump. The CMJ is performed starting from a standing position, then a quick movement of flexion and extension of the knees, and an immediate maximum vertical jump [22]. Before the start of the vertical jump tests, participants performed a warm-up on a cycle ergometer for 10 minutes. After the warm-up period, each subject was instructed in the proper way to perform each test. Before starting the test, a trial test was performed to verify that the participant had understood the instructions. Between every jump a rest period of 1 minute was set.

#### Anthropometry

Anthropometric data were obtained following the guidelines of The International Society for the Advancement of Kinanthropometry (ISAK) [23]. The weight was recorded with the subject barefoot and in underwear. The height is the distance from the vertex to the soles of the feet. It is measured with the subject standing in anatomical position and the occipital region, back, gluteal region and heels in contact with the height rod. The subject takes a deep breath at the time of measurement. The body mass index (BMI) was calculated by dividing weight in kilograms (kg) by height in meters squared (m^2^).

#### Kinematics Variables

Linear acceleration was measured along three orthogonal axes using the iPhone 4S inertial sensor, which incorporates a three-axis gyroscope, accelerometer and magnetometer. The mobile phone was attached to a belt and fixed at L5-S1 level. Data were obtained for analysis through SensorLog, available as an Apple iPhone app. The recording rate was set at 30 milliseconds. The recordings were stored in the internal memory of the mobile phone and were then sent via email for off-line processing. A previous study [24] showed that the mobile phone (iPhone) accelerometer was accurate and precise compared to a gold standard, with an intra-class correlation coefficient (*r* ^2^>0.98). The mobile phone accelerometer showed excellent sequential increases with increased walking velocity and energy expenditure (*r* ^2^>0.9). An accelerometer embedded into a mobile phone was accurate and reliable in measuring and quantifying physical activity in the laboratory setting [24].

#### Jump Measuring

The height and flight time were evaluated through the contact mat Globus Ergojump Thesys in CMJ with arm swing and SJ tests. The Globus Ergojump contact mat was validated in a prior study [12].

The CMJ test is performed with the subject starting from a standing position on the contact mat. A quick flexion and extension of the knee joint with minimal stops between eccentric and concentric phases is performed. The participant can swing his arms to propel himself. The legs should be kept in extension from take-off to landing. In a specific study to determine the reliability of different countermovement tests, an intraclass correlation coefficient (ICC) of 0.88 for the CMJ was determined [25].

For the SJ test, the participant is placed from the vertical position with hands on hips and with knees in flexion position of 90 degrees. Following the indication of the examiner, the subject performs a boost without any countermovement trying to achieve the maximum height in a vertical jump keeping the lower limbs in extension after take-off to landing [12].

The contact mat records flight time in seconds and the height reached in centimeters. Three repetitions of each test were conducted with more than one-minute rest between each.

#### Maximum Isotonic Strength of the Knee Extensors

Isotonic muscle strength of the knee extensors was evaluated by bilateral dynamometry through the digital manual dynamometer POWERTRACK (JtechMedical). This tool incorporates a load cell affixed to the distal end of the leg of the subject. The dynamometer has a digital display that shows the force applied to the load cell in Newtons and records the peak in each attempt. The validity of this dynamometer has been demonstrated, with an ICCs ranging from 0.72 to 0.85 [26].

The participant is placed in a sitting position on a stretcher, his hands resting on his legs and feet hanging off the ground. The examiner places one hand to stabilize the subject’s leg and the other hand to support the load cell on the subject’s distal third tibia. Starting from 90° knee flexion, the subject performs a knee extension resisted by the examiner with the load cell. A full extension must be avoided, with the knee flexion reaching 5°. The maximum peak force is recorded in the digital dynamometer. The test was performed three times for each subject, with a 2-minute break between tests; the highest value was taken.

### Data Processing

An off-line analysis was guided to obtain kinematic information from the accelerometer for each subject, in each trial, in the SJ and CMJ test. In this study, the mean and standard deviation was obtained from the maximum peak and minimum peak of accelerations in the three axes of movements (*x*, *y* and *z*). Furthermore, the mean and standard deviation from maximum peak and minimum peak from the resultant vector (RV) accelerations [RV = √ (*x* ^2^ + *y* ^2^ + *z* ^2^)] was obtained.

### Statistical Analysis

To analyze the results, a database was created from the information gathered from the participants, the inertial sensor variables, the jump test variables and maximum isotonic strength of the knee extensors variables. The Kolmogorov-Smirnov test was used as determined by the variables normality of distribution. Descriptive statistics were performed with measures of central tendency and dispersion of the variables studied. Analysis was performed with SPSS Version 20.0 (SPSS Inc, Chicago, IL, USA).

##  Results

The Kolmogorov-Smirnov demonstrated that the distribution of the sample by gender was non-normal. Table 2 summarizes the acceleration-based measures, the jump test measures and maximum isotonic strength of the knee extensors measures in the SJ and CMJ jump test.

**Table 2:** Acceleration-based, jump test values and maximum isotonic strength in the SJ and CMJ (N=81 jumps).

		Mean(SD)
**Accelerometer SJ**		
	Max acc X (m/s^2^)	0.585 (.449)
	Min acc X (m/s^2^)	-0.549 (.436)
	Max acc Y (m/s^2^)	1.851 (.629)
	Min acc Y (m/s^2^)	-0.087 (.239)
	Max acc Z (m/s^2^)	0.844 (.493)
	Min acc Z (m/s^2^)	-1.211 (.567)
	Max acc RV (m/s^2^)	2.202 (.679)
	Min acc RV (m/s^2^)	0.005 (.003)
**Accelerometer CMJ**		
	Max acc X (m/s^2^)	0.819 (.5385)
	Min acc X (m/s^2^)	-0.792 (.501)
	Max acc Y (m/s^2^)	2.036 (.645)
	Min acc Y (m/s^2^)	-1.158 (.076)
	Max acc Z (m/s^2^)	1.046 (.549)
	Min acc Z (m/s^2^)	-1.489 (.446)
	Max acc RV (m/s^2^)	2.472 (.631)
	Min acc RV (m/s^2^)	0.004 (.002)
**Jump Test Mat**		
	Jump height SJ (m)	0.223 (.076)
	Jump time SJ (s)	0.419 (.077)
	Jump height CMJ (m)	0.329 (.099)
	Jump time CMJ (s)	0.511 (.088)
**Dynamometry**		
	Right dynamometry (N)	251.92 (53.029)
	Left dynamometry (N)	234.96 (45.846)

##  Discussion

### Principal Results

In the present study the kinematic variables derived from acceleration through the inertial sensor of an iPhone 4S, dynamometry of lower limbs with a handheld dynamometer, and the height and flight time with a contact mat were described in vertical jump tests from a cohort of young healthy subjects, aged between 18 and 35 years. The development of the execution in SJ and CMJ vertical jump tests is described, examined and identified under acceleration variables obtained with the mobile phone.

### Comparison With Prior Work

To the best of our knowledge, and according to the literature reviewed so far, the present study is the first to describe and analyze the kinematic variables in vertical jump tests with the use of a mobile phone as the main instrument.

Previous studies have been found in literature that may be relevant to the present study, in which vertical jump tests were evaluated through inertial sensors [15-19,27,28]. However, none of them has instrumentalized jump tests with a mobile phone’s inertial sensor. Furthermore, most of these studies have focused on searching algorithms to identify jump height [15], and developing validations of accelerometers as vertical jump evaluators [16-19,27], rather than on the kinematic description of the jump tests through accelerometry variables. Only one of these studies [28] describes the accelerometic characteristics and analyses the acceleration curves during an SJ. However, acceleration features of SJ were not compared with any other type of jump test. On the other hand, the instrumentalization used was a uniaxial accelerometer and not a mobile phone’s triaxial accelerometer. The mobile phone’s triaxial accelerometer offers the advantage of getting variables of the three axes of motion (*x*, *y* and *z*), while the uniaxial accelerometer gives us variables of only one axis of movement. In addition, today, most mobile phones contain triaxial accelerometers, which are very accessible to most people, and the acquisition of another tool to analyze human motion through acceleration values would not be necessary using a mobile phone.

From accelerometry data obtained through the mobile phone, differences in values of minimum and maximum acceleration were identified between the two types of jump tests SJ and CMJ (see Table 2). Higher values of acceleration in the different axes of movement (*x*, *y* and *z*) in CMJ with respect to the SJ, may be explained by the countermovement produced in CMJ test causes greater movement synergies involving higher acceleration [29]. It can also be observed in the jump variables measured by the contact mat; higher mean values of jump height and jump time in the CMJ regarding SJ are shown (see Table 2).

**Figure 1:**
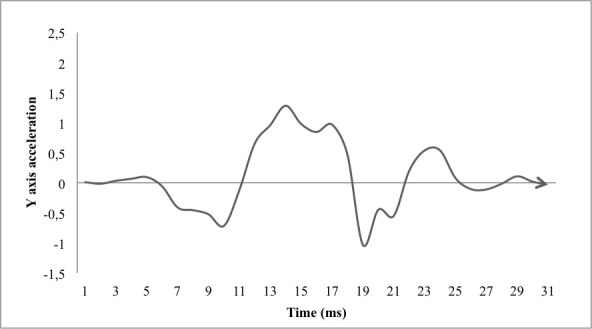
SJ Y axis acceleration graphic example.

**Figure 2:**
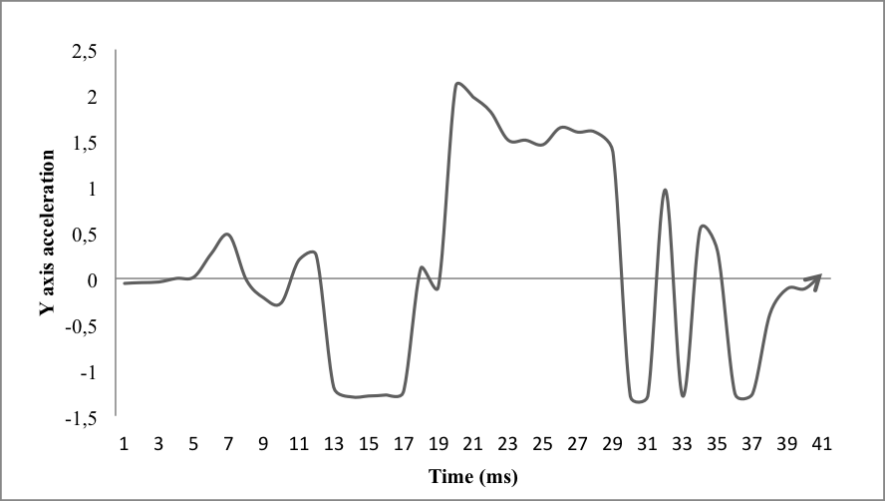
CMJ Y axis acceleration graphic example.

Specifically, the higher maximum acceleration values in the y-axis found in the CMJ are explained by the jump modality, as the countermovement allowed in it facilitates greater vertical impulse [29]. Furthermore, the fact that greater height is reached more easily at CMJ causes greater deceleration after the transition between the maximum peak height and landing; this results in higher minimum acceleration values along the y-axis with a remarkable difference between CMJ and SJ tests—the acceleration difference is the greatest between the two jumps. These perceived differences are consistent with the higher heights and flight times recorded by the contact mat from CMJ test and the known differences between CMJ and SJ tests [29]. Moreover, when analyzing the graphs of acceleration of SJ performed in this study, it has been observed that in the development of this type of jump, a negative acceleration on the y-axis (see Figure 1 and 2) is a common occurrence prior to the start of the upward positive acceleration curve that corresponds to the beginning of the upward thrust. Because the SJ technique must be performed without any countermovement, this could be explained by the possibility that the mobile phone's accelerometer detects small accelerations produced by short, quick countermovements made by the subject, and examiners who determine whether the execution of the jump has been correct, would not be able to detect them with their own eyes.

For acceleration in the z-axis, the differences found may also be explained by the influence of the transition between the eccentric and concentric phases occurring before take-off for CMJ because trunk flexo-extension movements occur, involving higher acceleration values in the z-axis [29].

Regarding the differences in the x-axis acceleration, it is conceivable that lateral displacements by the subject in the execution of both tests can be greater for the CMJ test, also because movement synergies in this jumping technique are implied, and higher flight times are achieved [29].

The differences between tests are also maintained for the resultant vector acceleration values, although less so. This can be justified by the integration of the acceleration signals in the different axes of motion to calculate the resulting vector.

### Limitations and Future Work

As a limitation in this study, we can mention the fact that there is no separation by gender analysis that would show differences between men and women in performing the jumps [30]. Therefore, future studies with a larger sample to allow a normal distribution of variables, adjusted by gender analysis, could be performed.

As a fortuitous finding, upon visual inspection of the y-axis acceleration graphic in the SJ tests, we observed a previous negative acceleration at the start of the upward curve of positive acceleration. This would be interesting research for future studies to analyze curves and globally analyze the mobile phone discriminating power between correct executions of vertical jump tests without countermovement based on trunk accelerometry.

### Conclusions

According to the results obtained in this study, we can conclude that the built-in iPhone 4S inertial sensor is able to measure acceleration variables for vertical jump tests SJ and CMJ in healthy young adults. The acceleration kinematics variables derivate from the mobile phone’s inertial sensor are higher in the CMJ test than the SJ test.

## Abbreviations

BMI: body mass index
CMJ: countermovement jump
ICC: intraclass correlation coefficient
ISAK: The International Society for the Advancement of Kinanthropometry
RV: resultant vector
SJ: squat jump
